# Genomic diversity of *Yersinia pestis* from Yunnan Province, China, implies a potential common ancestor as the source of two plague epidemics

**DOI:** 10.1038/s42003-023-05186-2

**Published:** 2023-08-15

**Authors:** Jingliang Qin, Yarong Wu, Liyuan Shi, Xiujuan Zuo, Xianglilan Zhang, Xiuwei Qian, Hang Fan, Yan Guo, Mengnan Cui, Haipeng Zhang, Fengyi Yang, Jinjiao Kong, Yajun Song, Ruifu Yang, Peng Wang, Yujun Cui

**Affiliations:** 1 State Key Laboratory of Pathogen and Biosecurity, Beijing Institute of Microbiology and Epidemiology, Beijing, China; 2 Yunnan Institute of Endemic Diseases Control and Prevention, Dali, China

**Keywords:** Bacterial evolution, Epidemiology, Comparative genomics, Bacterial genomics

## Abstract

Plague, caused by *Yersinia pestis*, is a zoonotic disease that can reemerge and cause outbreaks following decades of latency in natural plague foci. However, the genetic diversity and spread pattern of *Y. pestis* during these epidemic-silent cycles remain unclear. In this study, we analyze 356 *Y. pestis* genomes isolated between 1952 and 2016 in the Yunnan *Rattus tanezumi* plague focus, China, covering two epidemic-silent cycles. Through high-resolution genomic epidemiological analysis, we find that 96% of *Y. pestis* genomes belong to phylogroup 1.ORI2 and are subdivided into two sister clades (Sublineage1 and Sublineage2) characterized by different temporal-spatial distributions and genetic diversity. Most of the Sublineage1 strains are isolated from the first epidemic-silent cycle, while Sublineage2 strains are predominantly from the second cycle and revealing a west to east spread. The two sister clades evolved in parallel from a common ancestor and independently lead to two separate epidemics, confirming that the pathogen responsible for the second epidemic following the silent interval is not a descendant of the causative strain of the first epidemic. Our results provide a mechanism for defining epidemic-silent cycles in natural plague foci, which is valuable in the prevention and control of future plague outbreaks.

## Abstract (web summary)

Genome sequencing of Chinese *Yersinia pestis* isolates spanning more than 60 years and covering two epidemics and "silent” cycles in-between reveal the phylogeographic and evolutionary background of these plague cycles.

## Introduction

Plague is a deadly infectious disease caused by the gram-negative bacterium *Yersinia pestis*^1^. Three historic plague pandemics have caused over 160 million deaths worldwide^2^. The most recent of these pandemics, which began at the end of the 19th century in Hong Kong, China, and then spread to Africa, America, Oceania, and other parts of the world via maritime trade, lasted until the mid-20th century^3–5^. At present, plague remains a threat in many parts of the world and has been categorized by the World Health Organization since 2000 as a reemerging disease^6–8^.

Although *Y. pestis* can shape the natural plague foci under suitable ecological conditions, sylvatic plagues in these foci exhibit recurrent cycles of epidemic and silent periods with intervals ranging from several years to decades, rather than a single continuous epidemic^9,10^. During the epidemic period, animal plague usually occurs prior to human plague, whereas in the silent period, human plague disappears and animal plague fades out with occasional rodents detected carrying the F1 antibody or antigen around the corresponding foci^11,12^. However, there is a lack of knowledge regarding the genomic diversity of *Y. pestis* during these epidemic-silent intervals. It is unknown whether the reactivation of plague foci is caused by invasion of a new *Y. pestis* strain from other populations or the awakening of offspring from the latent strain.

The Yunnan *Rattus tanezumi* plague focus (*R. tanezumi*, domestic rodent plague focus) is one of the 12 major natural plague foci in China^13^. Active animal plague surveillance in this plague focus has been conducted since 1951^14^, and two epidemic periods and two silent periods have been documented since 1950^15,16^. The first plague epidemic period (named Epidemic1) was observed from 1950 to 1956, during which a plague outbreak occurred in 12 counties/cities in western Yunnan, involving 2950 human cases and 633 deaths^16^. Since 1957, plague has been well controlled, with few *Y. pestis* strains, bacteriophages, and positive sera of animals observed within the investigation area^17^. However, after a silence of 25 years, which we named the Silent1 period (1957–1981), another epidemic wave (Epidemic2) emerged in animals and subsequently spread to humans, lasting until 2007. Since 2008, cases of plague have faded again and the natural plague focus has entered a new silent period (Silent2), which is ongoing to present^15,18^.

Continuous surveillance data of more than 60 years and density sampling of *Y. pestis* isolates in Yunnan provide us with an opportunity to investigate the dynamics of the pathogen’s genomic diversity during these epidemic-silent cycles. Here, we analyzed the whole genomes of 356 *Y. pestis* strains isolated between 1952 and 2016 in the Yunnan *R. tanezumi* plague focus and combined genomic analysis with epidemiological data to infer genomic diversity, spread, and transfer patterns of *Y. pestis* across epidemic-silent intervals.

## Results

### Sampling of *Y. pestis* in Yunnan Province spanning two epidemic-silent cycles

**Fig. 1:**
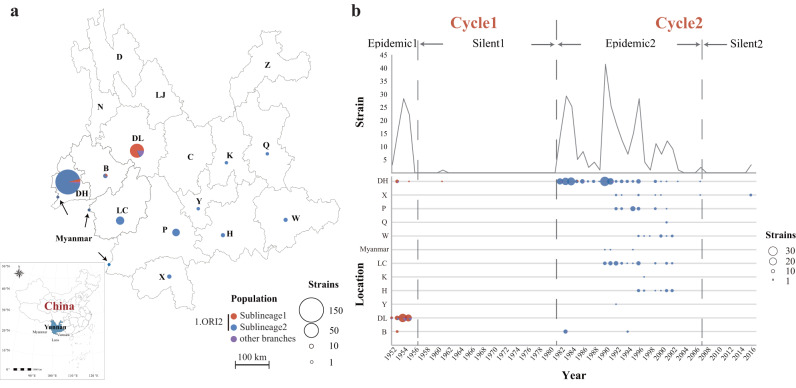
Distribution of *Y. pestis* strains isolated from Yunnan *R. tanezumi* plague focus between 1952 and 2016. **a** Geographic locations of Yunnan strains. Geographic abbreviations of Yunnan strain isolation locations are as follows: Dehong Dai-Jingpo Autonomous Prefecture (DH), Dali Bai Autonomous Prefecture (DL), Baoshan City (B), Lincang City (LC), Puer City (P), Honghe Hani-Yi Autonomous Prefecture (H), Yuxi City (Y), Xishuangbanna Dai Autonomous Prefecture (X), Kunming City (K), Qujing City (Q), Wenshan City (W). The pie chart area is proportional to the number of strains and colors of the pie indicate assigned phylogroups of strains. The map was generated using pandas (v1.4.2) and geopandas (v0.13.2) packages in Python3 based on the public geographical data downloaded from the Resource and Environment Science and Data Center of the Chinese Academy of Sciences (https://www.resdc.cn/). **b** Epidemic dynamics in Yunnan *R. tanezumi* plague foci. The isolation date of strain YN80071 was not clear and thus, was excluded from this analysis. The horizontal axis is the sampling date. The vertical axes represent the isolation location and number of strains. The plot is based on result in Supplementary Data 1.

In this study, 356 strains were isolated from 11 prefectures or cities in Yunnan Province, China, and Myanmar, near the China-Myanmar border, between 1952 and 2016, spanning two epidemic-silent cycles (Fig. 1a). Among them, 70 strains were isolated from the Epidemic1 period, one from Silent1, 282 from Epidemic2, and three from Silent2 (Table 1). To facilitate subsequent analysis, these strains were classified into Cycle1 (Epidemic1 and Silent1) and Cycle2 (Epidemic2 and Silent2) groups, according to their sampling dates (Fig. 1b). Specifically, we observed the greatest number of human cases during Cycle1 in 1954, and following a 25-year silent period, the plague epidemic reemerged in 1982, and then spread throughout the province, peaking in 1983, 1990, and 1996^15,16^.

**Table 1:** Phylogenetic positioning of 356 Yunnan strains.

Population	Yunnan *Rattus tanezumi* plague focus				Total
	The first plague epidemic period	The first plague silent period	The second plague epidemic period	The second plague silent period	
1.ORI2	60	1	279	3	343
Sublineage1^a^	58	1	1	0	60
Sublineage2^a^	2	0	277	3	282
YN86001^a^	0	0	1	0	1
1.IN3	5	0	0	0	5
1.IN2	3	0	0	0	3
1.IN1	1	0	0	0	1
2.MED3	0	0	1	0	1
2.ANT3	1	0	2	0	3
Total	70	1	282	3	356

^a^The assigned phylogroup/strain of 343 1.ORI2 strains.

### Genomic diversities of *Y. pestis* strains during the two cycles

**Fig. 2:**
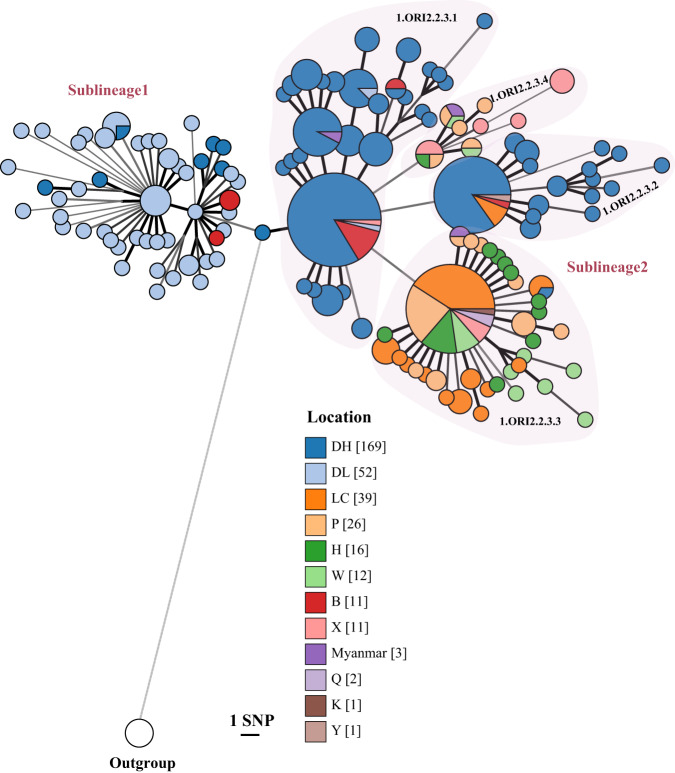
A minimal spanning tree reconstructed based on 263 SNPs identified in 343 strains of *Y. pestis* and colored according to sampling locations. Large and red text, Sublineage1 and Sublineage2; smaller letters, sub-clades (for example, 1.ORI2.2.3.1). Circle colors indicate the sampling locations.

**Fig. 3:**
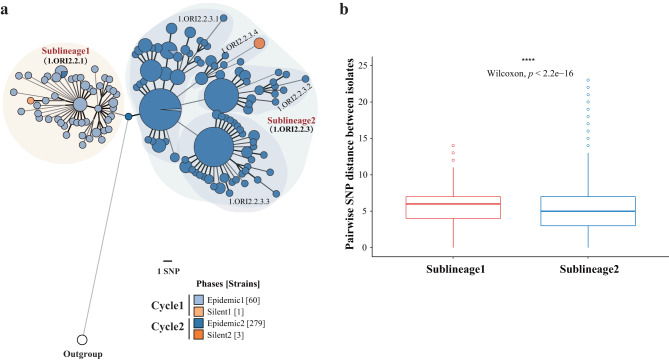
Population diversity of two plague epidemics in Yunnan Province. **a** A minimum spanning tree of 343 Yunnan strains (rooted with CO92 strain in 1.ORI1). Circle colors indicate the sampling date, and their size is proportional to the number of strains. Large, red text, Sublineage1 and 2; smaller letters, sub-clades (for example, 1.ORI2.2.3.1). Between nodes, numbers of single nucleotide polymorphisms (SNPs), apart from one SNP, are indicated by thick and thin black lines, respectively. **b** Pairwise SNP distance distributions within the two main clades. The center line of the boxplot is the median, bounds of the box are the first and third quartiles, circles are the outliers. The plot is based on result in Supplementary Data 3.

To infer the genomic diversities and population structure of *Y. pestis* in Yunnan during the two epidemic-silent cycles, we reconstructed the phylogeny of 356 isolates from the Yunnan *R. tanezumi* plague. Ninety-six percent of the strains (343 strains) were assigned to phylogroup 1.ORI2, which is associated with the third historic pandemic (Supplementary Fig. 1a). We excluded 13 strains scattered on other branches from further analysis and focused on the 343 strains in 1.ORI2 (Supplementary Data 1). The reconstructed phylogeny, based on 263 high-quality single nucleotide polymorphisms (SNPs), showed that these 343 1.ORI2 strains could be subdivided into two main clades, Sublineage1 and Sublineage2 (Figs. 2 and 3a). We discovered that Sublineage1 predominantly included strains from Cycle1 (59/60, 98.33%), characterized by three clade-specific SNPs, whereas most strains in the Sublineage2 clade came from Cycle2 (280/282, 99.29%), with one clade-specific SNP. Notably, four strains were isolated during two plague silent periods. Among them, one (Silent1, Cycle1) was located in the Sublineage1 clade and descended from Epidemic1 strains, and the other three (Silent2, Cycle2) were clustered together and located in the Sublineage2 clade and descended from Epidemic2 strains (Fig. 3a).

The phylogeny indicated that the two sublineages were sister clades because they split from a common ancestor, rather than one descending from the other. In addition, the two sublineages showed dissimilar genomic diversity. Although collected within a relatively short time interval, the pairwise SNP distance among strains in Sublineage1 (median = 6) was higher than those in Sublineage2 (median = 5; *p* < 0.001, Wilcoxon test) (Fig. 3b). Therefore, we inferred that the two plague epidemic periods in Yunnan were caused by two distinct lineages with a common ancestor and evolved in parallel.

### Spread and origin inference of *Y. pestis* in Yunnan plague epidemic

To infer the spread pattern of *Y. pestis* during the two epidemics in Yunnan, we integrated the information of phylogenetic relationships, sampling dates, and geographic distribution of the 343 *Y. pestis* strains. Phylogeographic analysis revealed that strains of Cycle1, mostly corresponding to Sublineage1, were distributed in western Yunnan, including Dehong Dai-Jingpo Autonomous Prefecture (DH), the Dali Bai Autonomous Prefecture (DL), and Baoshan City (B) (Figs. 1 and 2). Most strains from Cycle1 were isolated from DL (52/61, 85.25%); only 14.75% were from DH and B, suggesting that cross-region transmission events occurred among these regions during the Epidemic1 period.

**Fig. 4:**
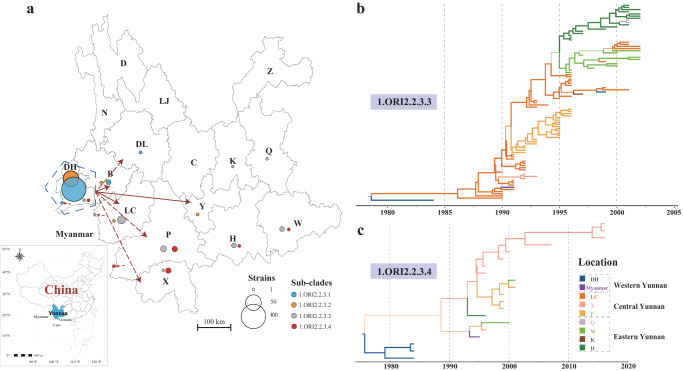
Transmission of Sublineage2 strains. **a** Geographic source of Sublineage2 strains. Spread routes are indicated by red lines with an arrow. Solid lines indicate direct Yunnan spread event routes based on phylogeographic analysis, while dotted lines indicate the possible or indirect spread event routes. The blue pentagon indicates the possible origin region of plague epidemics in Yunnan, which encompasses the most diverse strains. Filled circles represent numbers of strains whose groupings are indicated by colors (see legend at the right). Maximum clade credibility phylogenetic trees of 1.ORI2.2.3.3 (**b**) and 1.ORI2.2.3.4 (**c**) strains. Branch lengths are scaled to years. Branch colors indicate the sampling locations. Branch widths represent posterior probability value.

Sublineage2, including >99.29% of Cycle2 strains, can be further subdivided into four sub-clades (1.ORI2.2.3.1–1.ORI2.2.3.4) (Figs. 2 and 3a). Strains of the most basal lineage (1.ORI2.2.3.1) were mainly distributed in western Yunnan (Figs. 2 and 4a); 1.ORI2.2.3.2 strains were distributed in western and central Yunnan, 1.ORI2.2.3.3 strains were distributed in western, central, southern, and eastern Yunnan with a relatively shorter sampling time interval but a larger geographical span than the former two lineages; 1.ORI2.2.3.4, had a wide geographical range similar to that of 1.ORI2.2.3.3 (Figs. 2 and 4a). Bayesian-based phylogeographic analysis showed that the two widely distributed lineages, 1.ORI2.2.3.3 and 1.ORI2.2.3.4, exhibited a transmission trend from west to east (Fig. 4b, c). Additionally, most of the strains located on the deep branches were isolated from DH, a prefecture located in western Yunnan bordering northern Myanmar, where the plague epidemic lasted for the longest period. Thus, we inferred that the Epidemic2 plague, might have originated from DH Prefecture or its bordering countries, and then spread from the western to the central, southern, and eastern regions of Yunnan.

To further investigate whether the plague epidemic in Yunnan was caused by strains introduced from other countries, we collected 470 publicly available genomes (Supplementary Data 2) and used them to build a phylogeny with the newly sequenced genomes from this study (Supplementary Data 1). The results indicated that 1.ORI2 included 350 Yunnan strains, one Myanmar isolate, five Vietnam isolates, 57 South America isolates, one Zimbabwe isolate, and one Guangxi isolate from China (Supplementary Figs. 1a and 2). A high-resolution phylogenetic tree, containing only 1.ORI2 strains showed that the five Vietnam strains^19,20^, isolated in 1962 (Saigon-Nhatraung-62-3), 1967 (140-Dalat), 1986 (P-13273, P-13272), and 1988 (P-14709) were grouped together and formed a distinct clade (Supplementary Figs. 1b and 2, Supplementary Data 2). The Vietnam clade is embedded within the Yunnan clade in the overall phylogeny. The sampling dates of the five Vietnam strains correspond with the Silent1 and Epidemic2 periods in Yunnan, leading us to hypothesize that the introduction to Vietnam likely originated from Yunnan. However, due to the limited sampling in Southeast Asian countries, it is also conceivable that the common ancestor for both the Vietnam clade and Yunnan’s Sublineage1 and Sublineage2 might have originated elsewhere in Southeast Asia.

## Discussion

Epidemiological evidence indicates that plague in a natural focus is characterized by periodic alternations between epidemics and silence. Normally, plague outbreaks cause a population decline of local susceptible hosts so the population size falls below the threshold to maintain the epidemic, which then leads to the silence phase of plague in the focus. Following years or decades of recovery, the number of *Y. pestis*, hosts, and vectors reaches a dynamic balance again, rodent plague epizootics reemerge and may spillover to cause human epidemics^21^. Two hypothetical sources of pathogens may be related to the reactivation of natural plague foci. One hypothesis (Hypothesis1) holds that an ancestral-descendant connection exists between the two cycles. *Y. pestis* is preserved in individual hosts and vectors, or in the environment, such as soil, during the silent period, and its offspring can cause outbreaks again under suitable ecological conditions^22–25^. The second possibility (Hypothesis2) is that the introduction of new *Y. pestis* strains from other populations lead to the resurgence of plague in a specific focus^10,26^. This can occur through various scenarios, such as the rise of a distinct local population, spillover from an adjacent plague focus, or the introduction of a strain from a distant plague focus.

The long-lasting epidemiological surveillance of the Yunnan *R. tanezumi* plague provides an ideal sample set to test the above assumptions. The surveillance here indicated that between 1952 and 2016, it experienced two epidemic periods and two silent periods (Fig. 1b). *Y. pestis* strains were isolated annually from all epidemic regions in Yunnan over the past 60 years of surveillance. Through genomic and epidemiological analyses of 356 Yunnan strains, we found that, in general, *Y. pestis* strains from Cycle1 (including Epidemic1 and Silent1) and Cycle2 (including Epidemic2 and Silent2) could be subdivided into two sister clades: Sublineage1 (including 98.33% of Cycle1 strains) and Sublineage2 (including 99.29% of Cycle2 strains), respectively (Fig. 3a). These two clades evolved in parallel after splitting from a common ancestor and did not descend from one another. Of note, the pairwise SNP distance of Sublineage1 were relatively higher than that of Sublineage2, which indicates different patterns of genetic diversity between the two cycles. Taken together, both lines of evidence from perspectives of population structures and distribution patterns of genome-wide variations allow us to reject Hypothesis1 and to accept that Hypothesis2 is more plausible, that is, the Cycle1 plague and Cycle2 plague in Yunnan were caused by two independently evolved populations of *Y. pestis*.

We also inferred the spread pattern and possible origin of *Y. pestis* in Yunnan during the two epidemics. Phylogeographic analysis revealed an apparent transmission trend from west to east in Epidemic2. This trend was supported by two widely distributed sub-clades of Sublineage2, 1.ORI2.2.3.3 and 1.ORI2.2.3.4. Although strains from the other two sub-clades of Sublineage2 were confined within western and central Yunnan, in particular strains of the most basal lineage 1.ORI2.2.3.1 were predominantly distributed in western Yunnan, which does not contradict the spread trend of Epidemic2 from west to east. Unfortunately, due to uneven sampling coverage, with only 8.57% (*n* = 6) DH strains collected in Epidemic1, no obvious pattern could be summarized for spread during this period.

From the phylogeny, we observed that the deeply branched strains of all four sub-clades of Sublineage2 were predominantly isolated from DH (Fig. 2). DH is the most stubborn plague focus in Yunnan Province, which has the longest epidemic period and highest fatality rate^27^. Epidemiological data also indicated that DH was the area where plague first emerged and last disappeared during Epidemic2, and *Y. pestis* strains could be isolated in this region throughout the epidemic. Given that DH is a prefecture situated on the western edge of Yunnan Province and bordering northern Myanmar (Fig. 1a)^28^, it is plausible that Epidemic2 may have originated from DH Prefecture or its neighboring countries. However, due to limited sampling of *Y. pestis* strains in DH during Cycle1, there is insufficient to confirm that DH is the origin of both rounds of the Yunnan *R. tanezumi* plague epidemics. Considering that DH is an important port for China’s trade with Myanmar and other countries in Southeast and South Asia, it is necessary to further strengthen the surveillance of *Y. pestis* among animals in this region to prevent future human plague.

Epidemic-silent cycles of plague have been reported in other countries, with the plague in Mahajanga, a northwestern coastal city in Madagascar, being well-studied^29^. Plague reemerged in Mahajanga from 1991 to 1999 after a silent period lasting over six decades since 1928^10,30^. Several studies suggest that the outbreaks in the 1990s were caused by the reintroduction of new *Y. pestis* populations from the Central Highlands, rather than a latent strain preserved in local wildlife or the environment^10,29,30^. This supports Hypothesis2 and rejects Hypothesis1, which is consistent with our speculation regarding the two cycles of Yunnan plague. The main difference might be that the two epidemics in Yunnan originated from sister populations with a common ancestor, likely existing in local or adjacent areas, while the plagues in Mahajanga resulted from population migrations from a distant plague focus. Furthermore, Gomez et al. found that the two reemerged outbreaks in Mahajanga during the 1990s (the first from 1991 to 1992 and the second from 1995 to 1999) were attributed to two independent migration events from the Central Highlands occurring in the early 1980s, a decade before the first human cases were reported. They propose that *Y. pestis* might survive in local wildlife before transmission to humans. This could also be the case for the Yunnan plagues, as human plague appeared four years later than the epizootic plague in Epidemic2^15,16^.

Our study has several limitations. First, a sampling bias should be noted. The severity of plague was comparable between DL and DH during the Epidemic1 period. However, because of staffing shortages and material and financial resources in the 1950s, Epidemic1 strains were rarely collected in DH. Thus, there is insufficient genomic evidence to infer the origin and spread pattern of Epidemic1. Together with insufficient sampling of *Y. pestis* strains in Southeast Asia, it is difficult to investigate the origin of Yunnan *R. tanezumi* plague epidemics. Second, although no significant genomic variations in the strains responsible for Cycle1 and Cycle2 were identified, a bacterial pathogenicity experiment is still needed to determine the difference between *Y. pestis* isolated from these two periods to further explore the mechanism of epidemic cycles. Finally, ecological and environmental dynamics in the niche, such as climate change, have been proven to be related to plague outbreaks^31,32^. A comprehensive analysis of eco-evolutionary dynamics should be conducted in the future to clarify the causes and mechanisms, leading to the resurgence of the plague epidemic.

In this study, we analyzed the genetic diversity and spread pattern of *Y. pestis* strains sampled over 60 years from the Yunnan *R. tanezumi* plague focus, which were involved in two epidemic-silent cycles. We found that the plague in Yunnan Province originated from DH or its adjacent countries and spread from west to east. Importantly, we found that the two plague epidemics in Yunnan were caused by two sister clades that evolved in parallel from a common ancestor and showed different genetic diversity. Our results provide robust genomic evidence that the second epidemic was led by a common ancestor, rather than a descendant of Epidemic1. A similar pattern might be the cause of epidemic-silent cycles in other natural plague foci globally, which needs further verification using a suitable dataset in the future.

## Methods

### Bacterial strains, sequencing, and assembly

A total of 826 *Y. pestis* genomes were used in this study. Among them, 470 global isolates were downloaded from The National Center for Biotechnology Information GenBank on October 19, 2020 (Supplementary Data 2) and 356 genomes were collected from the Yunnan *R. tanezumi* plague focus (Supplementary Data 1), including 175 genomes isolated from DH, and 181 newly sequenced genomes isolated from other regions of Yunnan.

The DNA of the newly sequenced strains was extracted using the QIAGEN DNeasy Blood & Tissue (No. 69506) kit (QIAGEN Shanghai, China). Whole-genome sequencing was performed on the Illumina X-Ten platform with a 150-bp paired-end sequencing library. An average of 500 Mb of clean data were generated for each strain. The reads were then assembled using SPAdes^33^.

### SNP calling

All assemblies were compared to the reference genome, *Y. pestis* CO92 (1.ORI1 phylogroup strain) (accession no. NC_003143.1) using MUMmer (v3.0)^34^ to generate base alignments and identify SNPs. Sequencing reads were mapped to the reference to evaluate SNP accuracy for each strain using BWA (v0.7)^35,36^ and GATK (v3.8)^37,38^. Only high-quality SNPs, with base quality >20 (i.e., the accuracy of a particular base within mapped reads is higher than 99%), supported by >10 reads, and present in the core genome shared by at least 95% of the total strains, were retained for further analysis. SNPs located in repetitive regions were removed from the SNP dataset.

### Phylogenetic analyses

A total of 3851 SNPs were identified in the 826 *Y. pestis* strains, which were concatenated to construct a maximum likelihood tree using IQ-TREE (v1.6)^39^ under the Generalized Time Reversible (GTR) model with 100 bootstrap replicates to assess tree topology support^40^. Phylogenic analysis showed that 96.35% (343/356) of the Yunnan *Y. pestis* strains belonged to phylogroup 1.ORI2.

To obtain a reliable topology with a high resolution of 1.ORI2 strains, we recalled the SNPs for the 343 Yunnan strains within this clade based on the same pipeline. Finally, 263 high-quality and non-homoplastic SNPs were identified and imported into Applied Maths Bionumerics 6.6 software to build a minimum spanning (MS) tree with hypothetical intermediate nodes for all 343 *Y. pestis* strains, with CO92 as the outgroup.

### Bayesian-based phylogeographic analysis

TempEst (v1.5)^41^ was employed to estimate the temporal signal before utilizing the concatenated sequences of SNPs, sampling locations, and dates of strains for discrete phylogeographic analysis in the BEAST software (v1.10)^40^ to speculate the spread routes of *Y. pestis* in Yunnan. We selected the GTR+γ substitution model, uncorrelated relaxed clock for substitution rate, constant population size, and Bayesian skyline for tree priors to perform Bayesian analysis. For each analysis, three independent Markov Chain Monte Carlo chains of 10^7^ cycles for sub-clade 1.ORI2.2.3.3, and 10^6^ for sub-clade 1.ORI2.2.3.4 were carried out, respectively, and then combined using LogCombiner (v2.6). Tracer (v1.7)^42^ was used to access the convergence and ensure that the effective sample sizes of all relevant parameters were above 200^43^. TreeAnnotator (v1.10) was used to generate a maximum clade credibility (MCC) tree, with the first 10% of the states excluded as burn-in. The MCC tree was visualized and modified using FigTree (v1.4) (http://tree.bio.ed.ac.uk/software/figtree/).

### Reporting summary

Further information on research design is available in the Nature Portfolio Reporting Summary linked to this article.

### Supplementary information

Supplementary Information

Description of Additional Supplementary Files

Supplementary Data 1

Supplementary Data 2

Supplementary Data 3

Reporting Summary

## Data Availability

Raw sequence data and the newly sequenced genomes of this work were submitted to NCBI under BioProject ID PRJNA910854. Additional source data for Figs. 1b and 3b are provided as Supplementary Data 1 and 3. Background information of 470 public genomes used in this work can be found in Supplementary Data 2.
