# Supplementary material for: Genomic diversity of Yersinia pestis from Yunnan Province, China, implies a potential common ancestor as the source of two plague epidemics
Source: Commun Biol. 2023 Aug 15;6:847. doi: 10.1038/s42003-023-05186-2 (PMC10427647; doi:10.1038/s42003-023-05186-2)
Supplement: Supplementary file 1 — Supplementary Information [file 42003_2023_5186_MOESM1_ESM.pdf]

Genomic diversity of *Yersinia pestis* from Yunnan Province, China, implies a potential common ancestor as the source of two plague epidemics

Jingliang Qin[1#], Yarong Wu[1#], Liyuan Shi[2#], Xiujuan Zuo[1], Xianglilan Zhang[1], Xiuwei Qian[1], Hang Fan[1], Yan Guo[1], Mengnan Cui[1], Haipeng Zhang[2], Fengyi Yang[2], Jinjiao Kong[2], Yajun Song[1], Ruifu Yang[1*], Peng Wang[2*] & Yujun Cui[1*]

Affiliations:

[1] State Key Laboratory of Pathogen and Biosecurity, Beijing Institute of Microbiology and Epidemiology, Beijing, China

[2] Yunnan Institute of Endemic Diseases Control and Prevention, Dali, China

#These authors contributed equally.

*Address correspondence to Ruifu Yang, ruifuyang@gmail.com, Peng Wang, wp030801@126.com, or Yujun Cui, cuiyujun.new@gmail.com

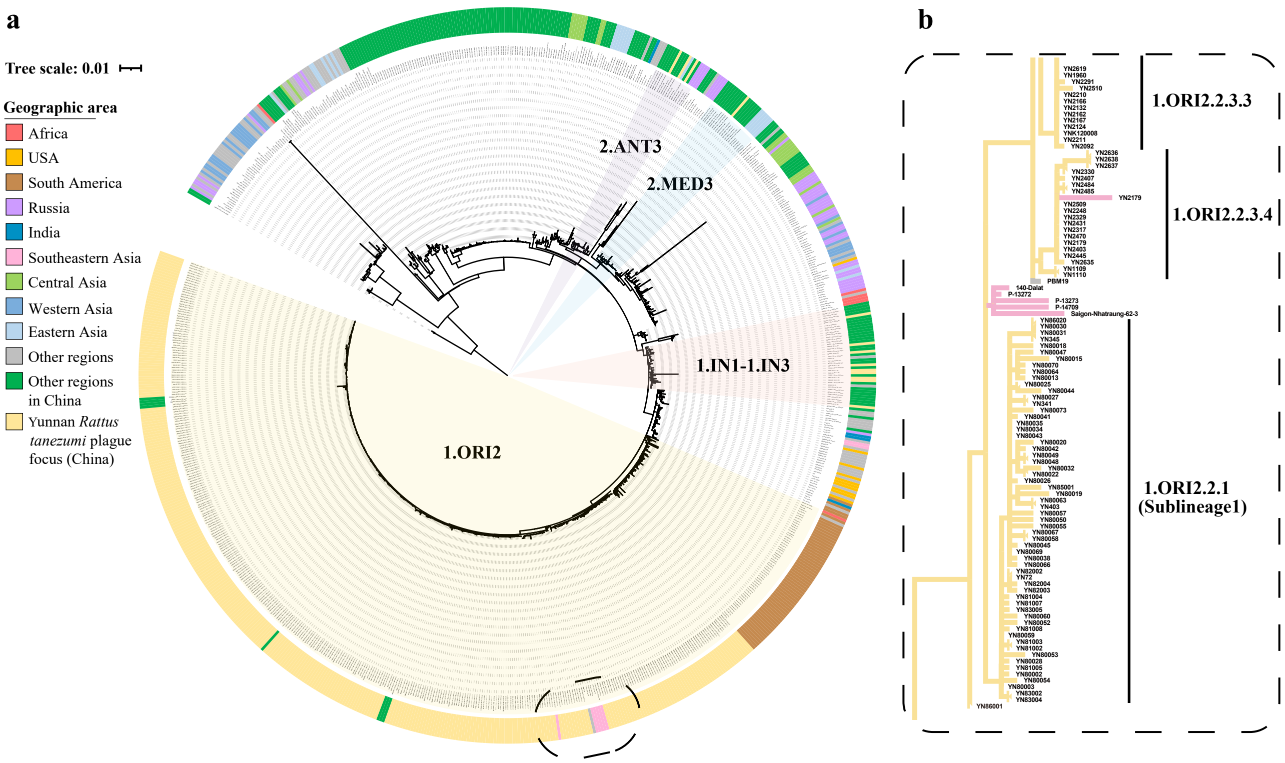

Supplementary Fig. 1 Phylogenetic tree of 826 *Yersinia pestis* genomes. a A maximum likelihood tree was generated based on a total of 3,851 single nucleotide polymorphisms. The outer ring represents the geographic locations. Phylogroups containing Yunnan strains were highlighted and labelled with corresponding names. **b** A fine-scale plot for 1.ORI2 strains marked by a dashed box in (**a**). It displays the topological structure of strains isolated from Southeastern Asia.

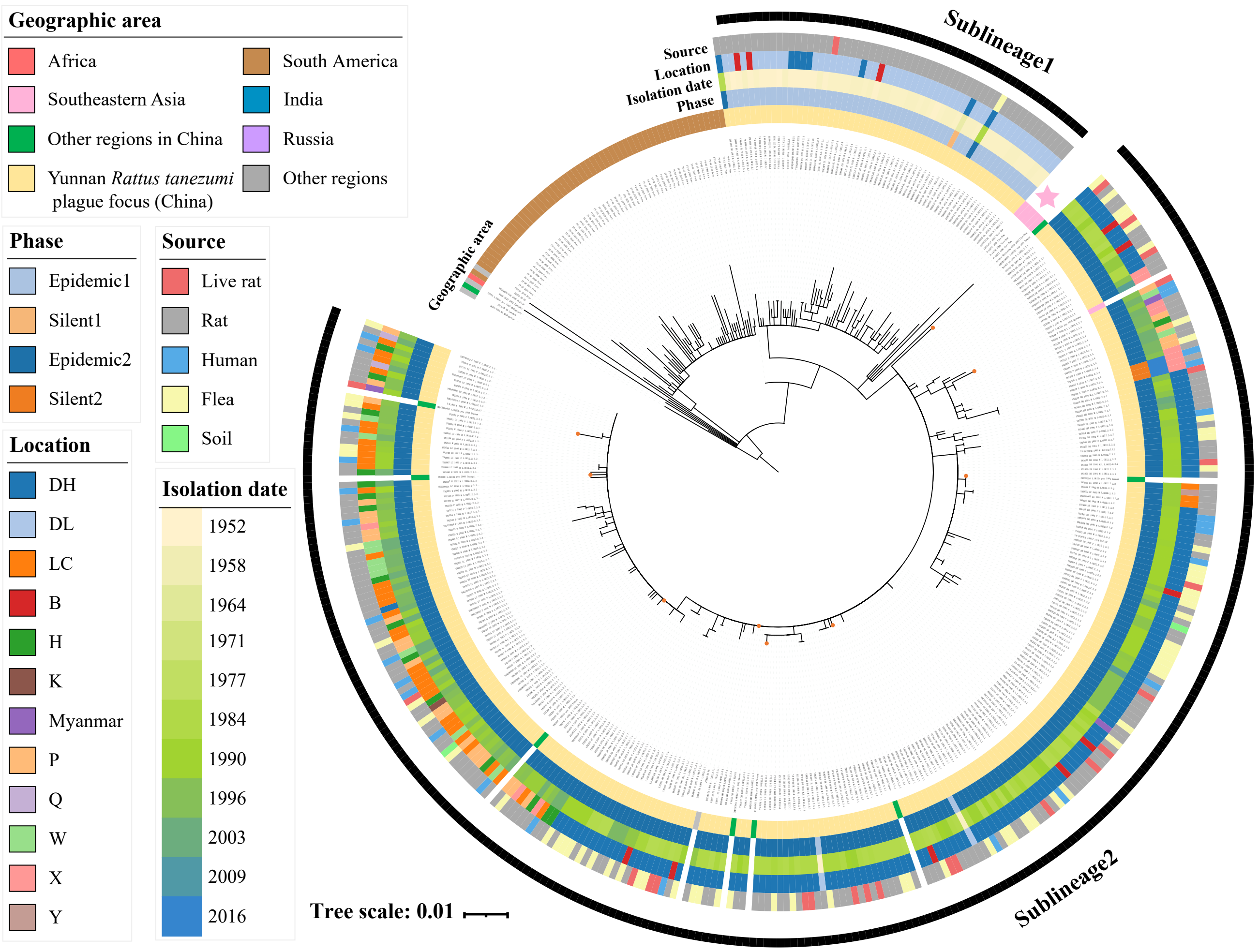

Supplementary Fig. 2 Maximum likelihood phylogenetic tree of 1.ORI2 strains. The following strain nomenclature was used: strain ID_location_isolation year_host_sub-clade name. Geographic abbreviations of Yunnan *R. tanezumi* plague focus strain isolation locations are as follows: Dehong Dai - Jingpo Autonomous Prefecture (DH), Dali Bai Autonomous Prefecture (DL), Baoshan City (B), Lincang City (LC), Puer City (P), Honghe Hani-Yi Autonomous Prefecture (H), Yuxi City (Y), Xishuangbanna Dai Autonomous Prefecture (X), Kunming City (K), Qujing City (Q), Wenshan City (W). The colors of rings from inner to outer indicate the geographic area, phase, isolation data, location, source, and clade. The pink pentagon indicates the phylogenetic positioning of five Vietnam strains. The orange dots in node labeled represent the nine strains (1.ORI2a-i) sequenced in the 2013 PNAS paper[1].

Supplementary References

1 Cui, Y. *et al.* Historical variations in mutation rate in an epidemic pathogen, *Yersinia pestis*. *Proc Natl Acad Sci U S A* **110**, 577-582, doi:10.1073/pnas.1205750110 (2013).
