# Supplementary material for: Genomic diversity of Yersinia pestis from Yunnan Province, China, implies a potential common ancestor as the source of two plague epidemics
Source: Commun Biol. 2023 Aug 15;6:847. doi: 10.1038/s42003-023-05186-2 (PMC10427647; doi:10.1038/s42003-023-05186-2)
Supplement: Supplementary file 2 — Description of Additional Supplementary Files [file 42003_2023_5186_MOESM2_ESM.pdf]

Description of Additional Supplementary Files

File name: Supplementary Data 1

Description: Background information of 356 *Y. pestis* strains isolated in the Yunnan *Rattus tanezumi* plague focus.

File name: Supplementary Data 2

Description: Background information of 470 public genomes used in this work.

File name: Supplementary Data 3

Description: The source data of the main figures.
