# Supplementary material for: Genomic diversity of Yersinia pestis from Yunnan Province, China, implies a potential common ancestor as the source of two plague epidemics
Source: Commun Biol. 2023 Aug 15;6:847. doi: 10.1038/s42003-023-05186-2 (PMC10427647; doi:10.1038/s42003-023-05186-2)
Supplement: Supplementary file 6 — Reporting Summary [file 42003_2023_5186_MOESM6_ESM.pdf]

nature portfolio

Corresponding author(s): Ruifu Yang, Peng Wang, or Yujun Cui

Last updated by author(s): Jul 21, 2023

# Reporting Summary

Nature Portfolio wishes to improve the reproducibility of the work that we publish. This form provides structure for consistency and transparency in reporting. For further information on Nature Portfolio policies, see our Editorial Policies and the Editorial Policy Checklist.

## Statistics

For all statistical analyses, confirm that the following items are present in the figure legend, table legend, main text, or Methods section.

| n/a | Confirmed | |
|---|---|---|
| ☐ | ☒ | The exact sample size (*n*) for each experimental group/condition, given as a discrete number and unit of measurement |
| ☒ | ☐ | A statement on whether measurements were taken from distinct samples or whether the same sample was measured repeatedly |
| ☐ | ☒ | The statistical test(s) used AND whether they are one- or two-sided<br>*Only common tests should be described solely by name; describe more complex techniques in the Methods section.* |
| ☒ | ☐ | A description of all covariates tested |
| ☒ | ☐ | A description of any assumptions or corrections, such as tests of normality and adjustment for multiple comparisons |
| ☐ | ☒ | A full description of the statistical parameters including central tendency (e.g. means) or other basic estimates (e.g. regression coefficient) AND variation (e.g. standard deviation) or associated estimates of uncertainty (e.g. confidence intervals) |
| ☒ | ☐ | For null hypothesis testing, the test statistic (e.g. *F*, *t*, *r*) with confidence intervals, effect sizes, degrees of freedom and *P* value noted<br>*Give P values as exact values whenever suitable.* |
| ☐ | ☒ | For Bayesian analysis, information on the choice of priors and Markov chain Monte Carlo settings |
| ☒ | ☐ | For hierarchical and complex designs, identification of the appropriate level for tests and full reporting of outcomes |
| ☒ | ☐ | Estimates of effect sizes (e.g. Cohen's *d*, Pearson's *r*), indicating how they were calculated |

*Our web collection on statistics for biologists contains articles on many of the points above.*

## Software and code

Policy information about availability of computer code

| | |
|---|---|
| Data collection | Whole-genome sequencing was performed on the Illumina X-Ten platform |
| Data analysis | All the softwares used in the study are open access and publicly available, including MUMmer (v3.0), BWA (v0.7), GATK (v3.8), IQ-TREE (v1.6), Applied Maths Bionumerics 6.6, TempEst (v1.5), BEAST (v1.10), LogCombiner (v2.6), Tracer (v1.7), TreeAnnotator (v1.10), FigTree (v1.4), Prokka, Roary |

For manuscripts utilizing custom algorithms or software that are central to the research but not yet described in published literature, software must be made available to editors and reviewers. We strongly encourage code deposition in a community repository (e.g. GitHub). See the Nature Portfolio guidelines for submitting code & software for further information.

## Data

Policy information about availability of data

All manuscripts must include a data availability statement. This statement should provide the following information, where applicable:

- Accession codes, unique identifiers, or web links for publicly available datasets
- A description of any restrictions on data availability
- For clinical datasets or third party data, please ensure that the statement adheres to our policy

Raw sequence data and the newly sequenced genomes of this work were submitted to NCBI under BioProject ID PRJNA910854. Background information of sequenced isolate is listed in Supplementary Table 1.

## Human research participants

Policy information about studies involving human research participants and Sex and Gender in Research.

| | |
|---|---|
| Reporting on sex and gender | NA |
| Population characteristics | NA |
| Recruitment | NA |
| Ethics oversight | NA |

Note that full information on the approval of the study protocol must also be provided in the manuscript.

# Field-specific reporting

Please select the one below that is the best fit for your research. If you are not sure, read the appropriate sections before making your selection.

☐ Life sciences ☐ Behavioural & social sciences ☒ Ecological, evolutionary & environmental sciences

For a reference copy of the document with all sections, see nature.com/documents/nr-reporting-summary-flat.pdf

# Ecological, evolutionary & environmental sciences study design

All studies must disclose on these points even when the disclosure is negative.

| | |
|---|---|
| Study description | Through high-resolution genomic epidemiological analysis, we analyzed 356 Y. pestis genomes isolated between 1952 and 2016 in the Yunnan Rattus tanezumi plague focus, China, covering two epidemic-silent cycles. |
| Research sample | In this study, 356 strains were isolated from 12 cities in Yunnan Province, China, between 1952 and 2016, spanning two epidemic-silent cycles. Among them, 70 strains were isolated from the Epidemic1 period, one from Silent1, 282 from Epidemic2, and three from Silent2. |
| Sampling strategy | Strains were collected from different municipal regions of Yunnan by the local Centre for Disease Control and Prevention between 1952 and 2016 during routine surveillance and plague outbreaks, according to the standard protocol of the National Scheme of Plague Surveillance of China (http://www.gov.cn/yjgl/2005-08/30/content_28245.htm). |
| Data collection | Active animal plague surveillance in Yunnan Rattus tanezumi plague focus |
| Timing and spatial scale | 356 strains were isolated from 12 cities in Yunnan Province, China, between 1952 and 2016, spanning two epidemic-silent cycles (Fig. 1a). Among them, 70 strains were isolated from the Epidemic1 period, one from Silent1, 282 from Epidemic2, and three from Silent2 (Table 1). |
| Data exclusions | NA |
| Reproducibility | NA |
| Randomization | NA |
| Blinding | NA |

Did the study involve field work? ☐ Yes ☒ No

# Reporting for specific materials, systems and methods

We require information from authors about some types of materials, experimental systems and methods used in many studies. Here, indicate whether each material, system or method listed is relevant to your study. If you are not sure if a list item applies to your research, read the appropriate section before selecting a response.

## Materials & experimental systems

| n/a | Involved in the study |
|---|---|
| ☒ | ☐ Antibodies |
| ☒ | ☐ Eukaryotic cell lines |
| ☒ | ☐ Palaeontology and archaeology |
| ☒ | ☐ Animals and other organisms |
| ☒ | ☐ Clinical data |
| ☒ | ☐ Dual use research of concern |

## Methods

| n/a | Involved in the study |
|---|---|
| ☒ | ☐ ChIP-seq |
| ☒ | ☐ Flow cytometry |
| ☒ | ☐ MRI-based neuroimaging |
